# Sustainable Cellulose-Derived Organic Photonic Gels with Tunable and Dynamic Structural Color

**DOI:** 10.1021/acsnano.3c11432

**Published:** 2024-01-12

**Authors:** Luyao Huang, Xianzhe Zhang, Lin Deng, Ying Wang, Yongmin Liu, Hongli Zhu

**Affiliations:** † Department of Mechanical and Industrial Engineering, Northeastern University, 360 Huntington Avenue, Boston, Massachusetts 02115, United States; ‡ Department of Electrical and Computer Engineering, Northeastern University, 360 Huntington Avenue, Boston, Massachusetts 02115, United States

**Keywords:** chiral nematic structure, hydroxypropyl cellulose, pitch, polyethylene glycols, structural color

## Abstract

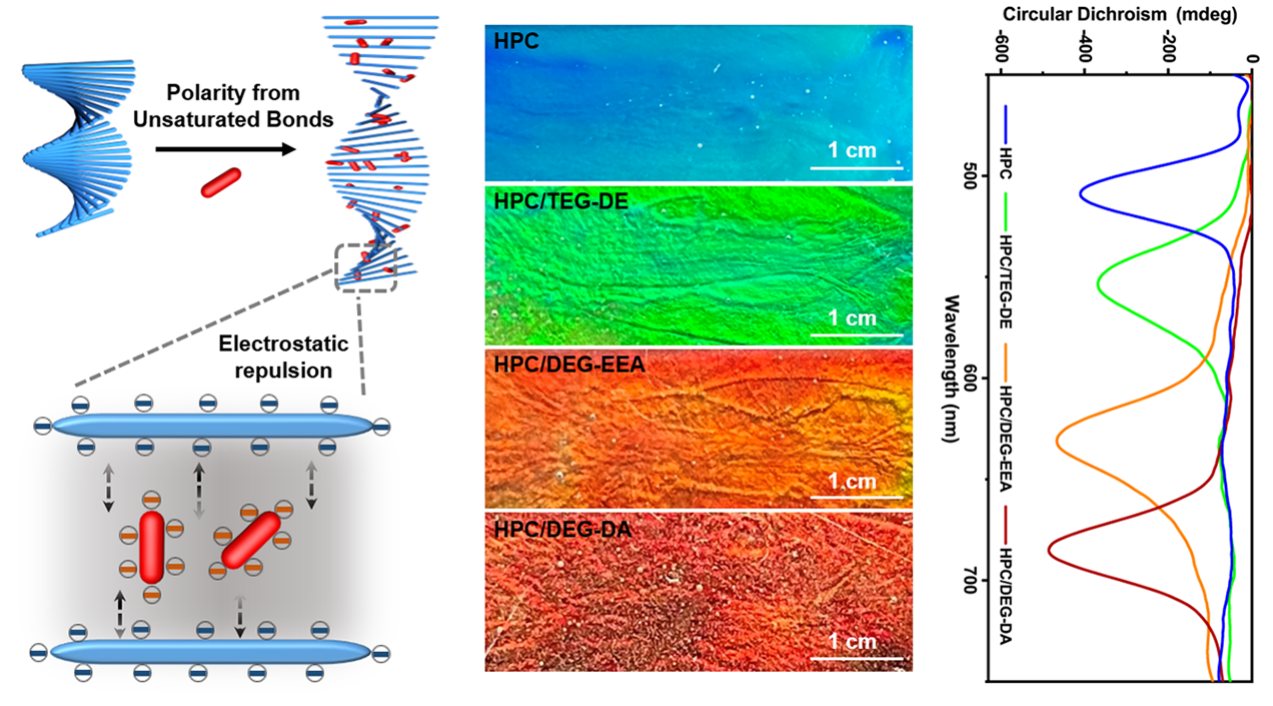

Structural color is a fascinating optical phenomenon arising from intricate light–matter interactions. Biological structural colors from natural polymers are invaluable in biomimetic design and sustainable construction. Here, we report a renewable, abundant, and biodegradable cellulose-derived organic gel that generates stable cholesteric liquid crystal structures with vivid structural colors. We construct the chromatic gel using a 68 wt % hydroxypropyl cellulose (HPC) matrix, incorporating distinct polyethylene glycol (PEG) guest molecules. The PEGs contain peculiar end groups with tailored polarity, allowing for precise positioning on the HPC helical backbone through electrostatic repulsion between the PEG and HPC chains. This preserves the HPC’s chiral nematic phase without being disrupted. We demonstrate that the PEGs’ polarity tunes the HPC gel’s reflective color. Additionally, gels with variable polarities are highly sensitive to temperature, pressure, and stretching, resulting in rapid, continuous, and reversible color changes. These exceptional dynamic traits establish the chiral nematic gel as an outstanding candidate for next-generation applications across displays, wearables, flexible electronics, health monitoring, and multifunctional sensors.

## Introduction

Structural color is produced by light interaction with natural or artificial photonic nanomaterials.^1−6^ In nature, organisms exhibit brilliant structural colors adapted through evolution for functions like camouflage, attraction, communication, and signaling.^7^ Structural color is highly saturated, highly persistent, and reversible.^8,9^ These attributes have motivated researchers to emulate natural photonic nanostructures using synthetic polymers and colloids. On the other hand, it is an intriguing strategy to directly utilize the materials existing in nature. Natural components facilitate photonic nanostructures with inherently low absorption and flexible mechanics. Renewability, sustainability, low cost, abundance, biocompatibility, and biodegradability are additional benefits.

As the most abundant natural polymer, regenerated cellulose holds great promise to meet critical characteristics for advanced materials.^10−12^ Hydroxypropyl cellulose (HPC), a cellulose derivative with hydroxypropyl substitutions on the glucose units, has gained particular attention.^13^ These modifications impart HPC solubility in both organic solvents^14^ and water,^15^ enabling diverse applications in pharmaceuticals, food, paper, ceramics, and more.^16−19^ Notably, HPC forms a chiral nematic liquid crystal phase at concentrations of 50–70 wt %.^15,20^ The helical stacking of molecularly oriented HPC layers can selectively reflect visible light if the periodic pitch matches the light wavelengths. This distinctive self-assembly has motivated extensive research into precisely controlling HPC’s optical properties.^15^ Multiple factors can tune the HPC periodic spacing and resultant reflected color, including temperature,^21−23^ surface interactions,^24^ mechanics,^25^ molecular weight,^26^ concentration,^27^ solvent environment,^28^ and incorporated species.^29^ However, high HPC viscosity poses processing challenges when targeting the crucial 50–70 wt % concentration range required for structural coloration.^30,31^

While prior works have shown chiral pitch tuning using spatial constraints or additive interactions, we hypothesize that direct intermolecular interaction between HPC and additives can also modulate the periodic helical structure. Here, we explore organic poly(ethylene glycol)s (PEGs) versus conventional inorganic salt additives. Initial evidence suggests organic PEGs/HPC may offer superior mechanical performance, including better stretchability and flexibility compared to those of inorganic salts. A natural polymer such as nanocellulose can also be the guest polymer, but consistent control over the molecular weight and structure of nanocellulose remains challenging due to its natural origin.^32−34^ This work aims to elucidate differences in interaction mechanisms between HPC and various guest molecules to provide a molecular-level understanding of structural color control.

Therefore, in order to test our hypothesis that small PEG molecules with different end groups can tune structural color, gels were prepared by adding PEGs into HPC. The chosen PEGs have varied polarities and electrostatically assemble to the HPC network. The degree of unsaturation at the PEG end groups correlate with the red shift in the structural color of the HPC gels. Based on this effect, we performed a surface charge study through zeta potential tests, microstructure analysis of HPC/PEG film via electron microscopy, and optical characterizations based on UV–vis spectroscopy, polarizing optical microscopy (POM), and circular dichroism (CD) measurements. The results indicate that the PEGs’ polarity increases HPC periodic structure spacing. Concurrently, we found that the HPC/PEG gels exhibit rapid, highly sensitive, and reversible color changes in response to temperature, pressure, and stretching.

## Results and Discussion

**Figure 1:**
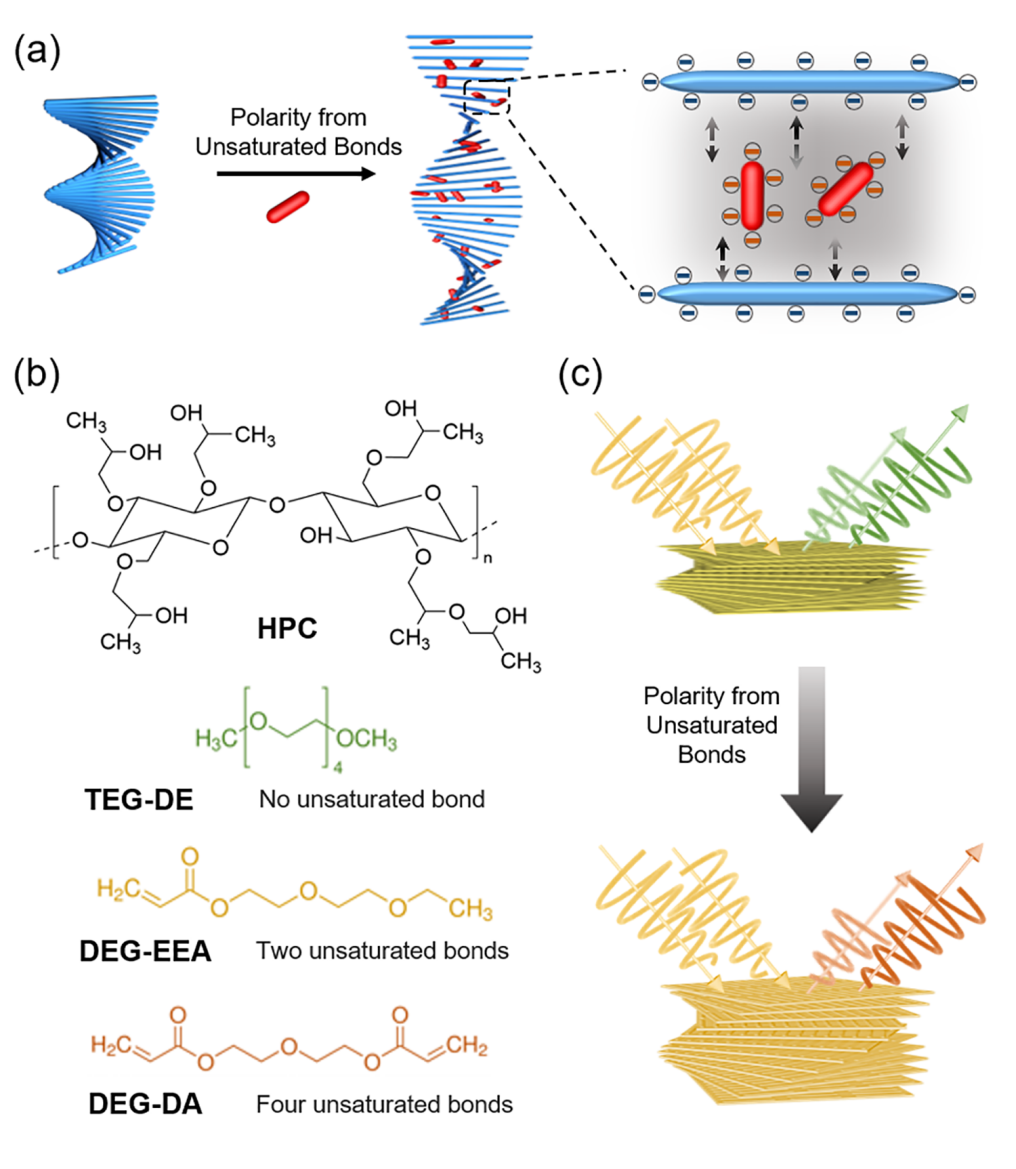
(a) Schematic representation of HPC gels undergoing structural changes. (b) Chemical molecular structures of each component used for organic photonic gel preparation. (c) Gels exhibiting varying periodic structures elicit modifications in circularly polarized light.

HPC forms a right-handed helical chiral nematic structure primarily due to its inherent molecular chirality and the intermolecular hydrogen bonding, which make the rigid, rod-like HPC molecules align in a specific, orderly structure that minimizes the system’s free energy. This self-assembly is further influenced by concentration, solvent effects, and dynamic principles.^35−37^ This work aims to investigate how interaction forces differ between the HPC and guest molecules of varying polarity. We had PEG uniformly mixed with HPC through high-speed mechanical mixing by a special dual-asymmetric centrifuge. After mixing, at the molecular level, HPC and PEG both possess negative surface charges resulting in electrostatic repulsion between the two components, as shown in Figure 1a. This repulsion leads to an alteration in the alignment period of the HPC polymer chains. To delve deeper into the underlying mechanisms governing the interaction forces that impact the cholesteric structure of HPC, we strategically selected three compounds for insertion into the HPC chains: tetraethylene glycol dimethyl ether (TEG-DE) with no unsaturated bond, diethylene glycol ethyl ether acrylate (DEG-EEA) with two unsaturated bonds, and diethylene glycol diacrylate (DEG-DA) with four unsaturated bonds. These compounds, as illustrated in Figure 1b, exhibit different degrees of unsaturation, characterized by no unsaturated bonds, two unsaturated bonds, and four unsaturated bonds, respectively. Furthermore, ester bonds possess a higher electronegativity compared to ether bonds or carbon–carbon double bonds.^38,39^ Consequently, we posited that the distinctive electronegativity intrinsic to the unsaturated bonds of PEG would engender marked cyclic volumetric expansion within the HPC polymers. This expansion, in our anticipation, would extend the optical path, ultimately leading to an alteration in the structural coloration, as depicted in Figure 1c. To streamline the process, we introduced 5 wt % PEG into HPC, thereby yielding four distinct electrostatic gels: HPC, HPC/TEG-DE, HPC/DEG-EEA, and HPC/DEG-DA.

**Figure 2:**
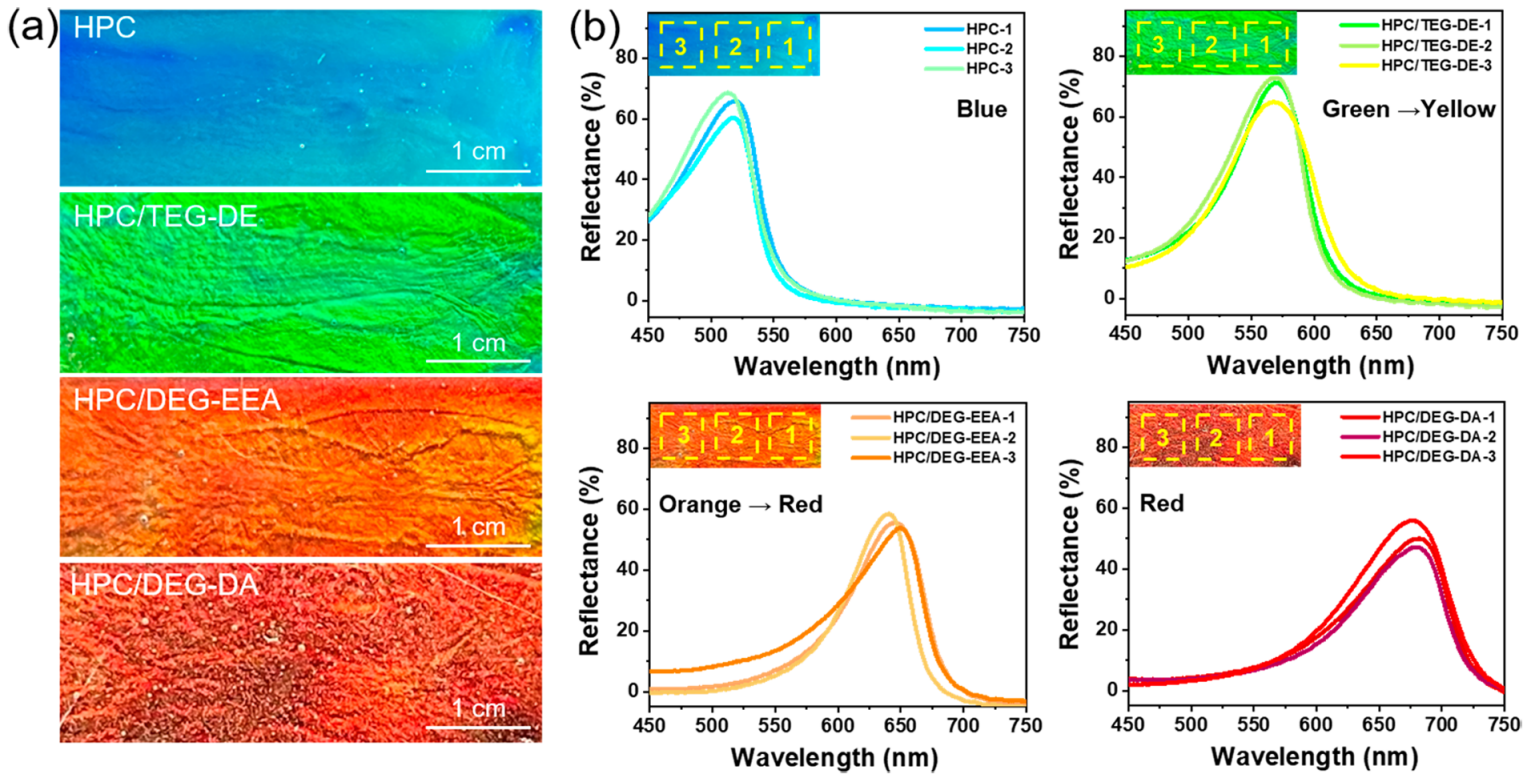
(a) Optical visible photographs of chiral liquid crystal gels with different PEG molecules at a viewing angle of 45° perpendicular to the surface of the film. (b) Reflectance spectra of HPC and PEGs composite gels measured by UV–vis.

The distinctive colors of the four gels, as shown in Figure 2a, confirm our hypothesis that the tailored PEG electronegativity precisely tunes the HPC periodicity. More specifically, the generated HPC/PEG gels exhibit rich colors by reflecting light through a cholesterol-type structure. A pure 68 wt % HPC gel was blue-green, but added PEGs tuned the color to a higher wavelength. The reflectance UV–vis spectra (Figure 2b) show that TEG-DE addition shifted the peak from 520 nm (blue) to 570 nm (yellowish green). With DEG-EEA and DEG-DA, further red shifts to 655 nm (orange) and 680 nm (red) occurred. Notably, the colors produced from the four samples span a very broad visible spectrum. The red-shift degree clearly correlates with the PEGs’ unsaturated bond number. DEG-EEA and DEG-DA with ester and carbon–carbon ends caused a greater red shift than saturated TEG-DE, while DEG-DA with four unsaturated bonds exceeds DEG-EEA with two unsaturated bonds. The color trend aligns with our hypothesis that PEGs with different end groups modulate the HPC gel color. We measured the wavelength peaks at three distinct positions on the left, middle, and right of each of the four gels. Subsequently, we conducted a statistical analysis on these wavelength peaks. The analysis of the wavelength peaks revealed that the composite gels, with the inclusion of PEGs, exhibited greater color homogeneity with a smaller standard deviation (Table S1).

**Figure 3:**
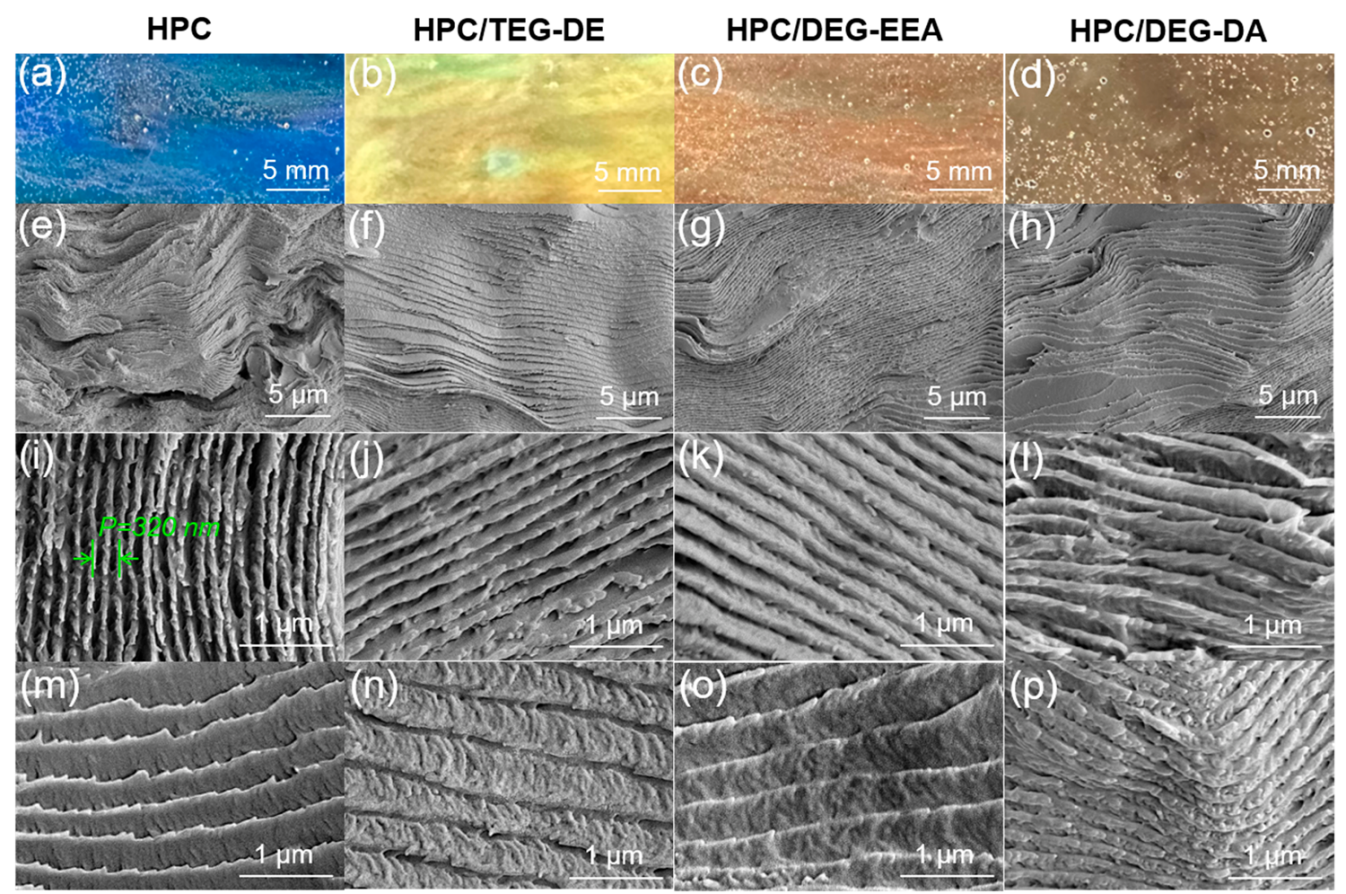
(a–d) Optical graphs of HPC/PEGs composite films. (e–h) Low-magnification cross-sectional SEM images. (i–l) Zoomed-in cross-sectional SEM images. (m–p) High-magnification oblique SEM images. Each row, from left to right, shows HPC, HPC/TEG-DE, HPC/DEG-EEA, and HPC/DEG-DA.

In order to elucidate the mechanism of the color response of the HPC gel structure to PEGs with different end groups, it is essential to investigate the impact of PEGs on the internal structure of HPC gels. To achieve this, we employed glutaraldehyde to cross-link the hydroxyl groups on the glucose units and immobilize the gel structure, thereby preparing HPC/PEG films. The optical graphs of the HPC/PEG composite film are presented in Figure 3a–d, revealing a spectrum of rainbow colors that shift from blue to red as the degree of PEG unsaturation rises. The colors of the HPC/PEGs composite films (Figure 3a–d) are not as vivid as in Figure 2a because glutaraldehyde was added for curing and the samples were dried for the subsequent scanning electron microscopy (SEM) characterization. Nonetheless, upon visual inspection of the photographs of the HPC/PEG composite films, a discernible spectrum of rainbow colors shifting from blue to red can still be observed as the degree of PEG unsaturation increases. SEM images obtained at low magnification clearly illustrate the ubiquitous cholesteric phase throughout the film’s thickness, as shown in Figure 3e,f. The cholesteric structure of the HPC film is uniformly aligned, which leads to the observation of uniform structural colors on the surface of the HPC film. The structural color is believed to be the result of Bragg reflection. Therefore, the peak wavelength in the reflectance spectra of solid HPC/PEGs composite films is related to the pitch length in the way given by
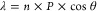
1Here λ is the peak wavelength of the reflected light, *n* is the average refractive index of the material that is approximately 1.5, *P* is the chiral nematic pitch length, and θ is the angle of incidence light. The refractive indices of the composite HPC/PEG gels and pure HPC gel are expected to be similar, given the small amount of added PEG (5% relative to the HPC mass).

The cross-sectional SEM images (Figure 3i–l) show that the films have regular parallel cholesterol layers with uniform spacing throughout the films. The distance between the two layers corresponds to the pitch, which was calculated to be approximately 320 nm for a pure HPC film (Figure 3i). TEG-DE readily penetrates the nematic layers due to similar polyhydroxyl groups and serves as a homogeneous HPC dispersant. Consequently, the repulsion between negative surface charges expands the interlayer distance and increases the peak wavelength of the reflectance spectra. The HPC nanorods balance this electrostatic repulsion and polyhydroxyl hydrogen bonding attraction, maintaining the helical arrangement. Specifically, polar TEG-DE incorporation expands the pitch from 320 to 380 nm. With unsaturated DEG-EEA and DEG-DA, the SEM images show further pitch increases to 440 and 460 nm, respectively, while the layered structure remains intact. This demonstrates that the PEG end group polarity promotes the spacing increase without disrupting the HPC equilibrium or cholesteric structure. Thus, PEG incorporation occupies free HPC volume, expanding the periodicity and red-shifting the structural color, consistent with our assumption. Excitingly, high-magnification SEM images (Figure 3m–p) reveal the right-handed helical nanorod structure twisted counterclockwise along the axis, with horizontal periodic layering preserved after PEG addition. This verifies that PEGs do not disrupt the intrinsic HPC cholesteric structure. Notably, the pitch follows the trend of increasing PEG unsaturation, agreeing with the associated gel color shifts.

**Figure 4:**
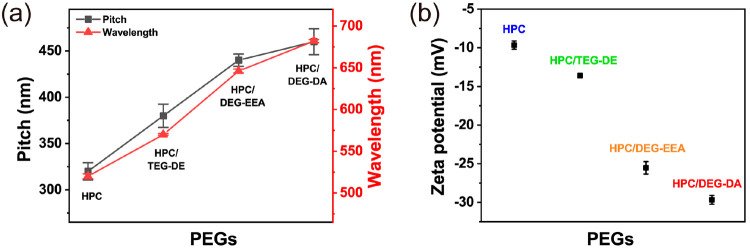
(a) SEM-measured chiral nematic pitch (black) and UV–vis-measured average reflected wavelength of the characteristic peaks (red) exhibited across films composed of HPC and PEG. (b) Zeta potentials of HPC composite solutions containing PEGs with different end groups.

The pitch values determined by SEM and the wavelengths at the peak reflectance measured from UV–vis spectroscopy (Figure 4a) generally follow eq 1. To visualize the distribution of pitches for different samples, we present a histogram in Figure S1 in the Supporting Information. The discrepancies between experimental results and eq 1 are mainly attributed to the SEM’s sensitivity to sample positioning. This sensitivity, in turn, is influenced by the angle at which the sample is oriented relative to the electron beam and the detection position. However, it is essential to note that these variations in SEM measurements do not diminish the established conclusion that an increase in PEG unsaturation corresponds to a proportional increase in HPC gel pitch. This trend remains consistent with the observed color pattern within the structure.

Investigating the HPC/PEG solutions provides insight into the influence and mechanism of PEG polarity in inducing ordered cholesterol arrangements and subsequent spacing expansion. At 5 wt % TEG-DE, the surface charge decreases from −9.7 to −13.6 mV (Figure 4b). This implies that PEGs increase HPC electrostatic repulsion, expanding the cholesteric spacing and red-shifting the structural color. Strikingly, replacing TEG-DE with DEG-EEA drops the charge to −25.5 mV, and further to −29.7 mV with DEG-DA. Thus, higher PEG electronegativity causes greater HPC/PEG repulsion. We conclude that the strong PEG negative charge produces repulsive HPC chain interactions, favoring uniform dispersion and stable gels. This in turn affects HPC self-assembly into an ordered chiral nematic phase. The increased PEG polarity elongates the helical pitch, consistent with the expanded periodicity observed by SEM. Overall, this elucidates how tailored PEG electronegativity controls the structural color of HPC gels.

**Figure 5:**
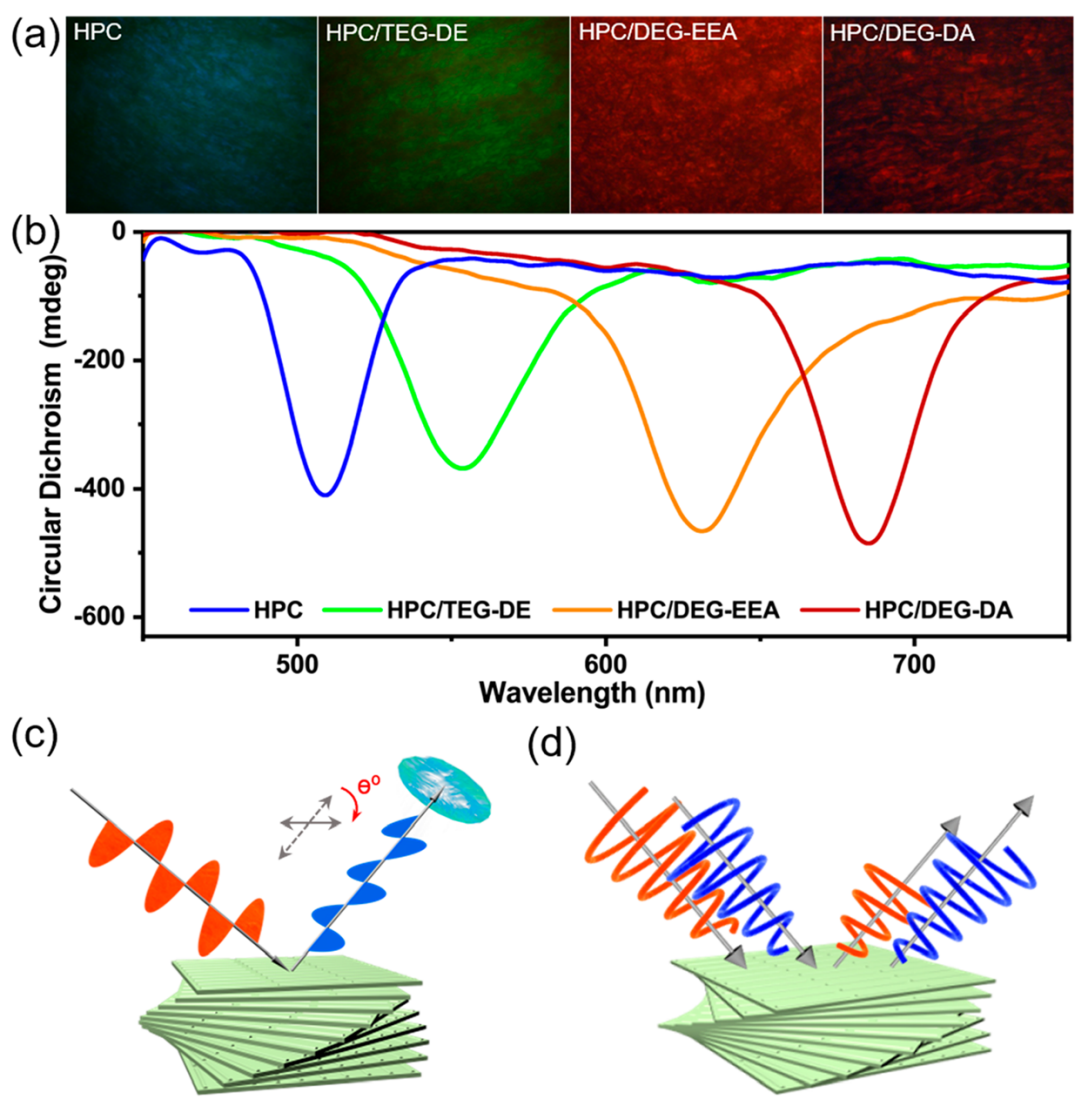
(a) POM images of HPC composite gels with different PEGs. (b) CD images of HPC composite gels with different PEGs. (c) Mechanism diagram of POM imaging of composite gels. (d) Mechanism diagram of CD measurement of composite gels.

Periodic structures with varied spacings were constructed using the repulsive interaction between HPC and PEG, quantified by the zeta potential. To characterize the chiral helical structure and interlayer spacing of HPC/PEGs, we performed POM and CD measurements (see Figure S2 and S3 for the home-built systems). Intermolecular forces induce molecular structural changes in HPC gels that drastically affect macroscopic polarized optical properties, as evidenced by POM. The samples were placed between two orthogonal polarizers, allowing for the detection of the gels’ polarization conversion ability. Figure 5a shows that all samples exhibit birefringent, chiral nematic fingerprint textures and can alter the polarization of the incident light. Additionally, as the unsaturated bond number of PEG increased, distinct birefringent color changes occurred, transitioning from shades of blue and green to orange, and eventually to red. This is because PEGs modify the cholesteric layer spacing of the composite gel and change the optical path through the heterogeneous material. Figure 5c illustrates how the polarization of light was altered after it interacted with the gels.

We also performed the CD experiments. The expression to retrieve CD spectra is given by^40^
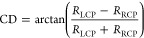
2where *R*_LCP_ and *R*_RCP_ are the reflectances of left-handed circularly polarized (LCP) and right-handed circularly polarized light (RCP) light through the sample, respectively. The reflectance of the CD was investigated, taking into account the limited absorption of light by the material, which showed agreement with prior detection. In the data derived from the CD experiments (Figure 5b), it is evident that all composite gels exhibited pronounced negative peaks on reflected CD spectra, substantiating the formation of right-handed cholesterol helices within the HPC gels (Figure 5c). It is noteworthy that the wavelength corresponding to the peak of the CD aligns with the observed coloration of the HPC/PEG gels, as documented through conventional optical microscopy and POM. Notably, even with the introduction of diverse PEGs, the composite gels maintained distinct, well-defined peaks. This observation suggests that the inclusion of PEGs primarily impacted the intermolecular chain spacing of the HPC polymers while concurrently preserving their inherent chiral attributes. As illustrated in Figure 5d, the difference in absorptions of different circularly polarized light after its interaction with the gel medium is depicted, resulting in a discernible wavelength shift in the CD spectra.

**Figure 6:**
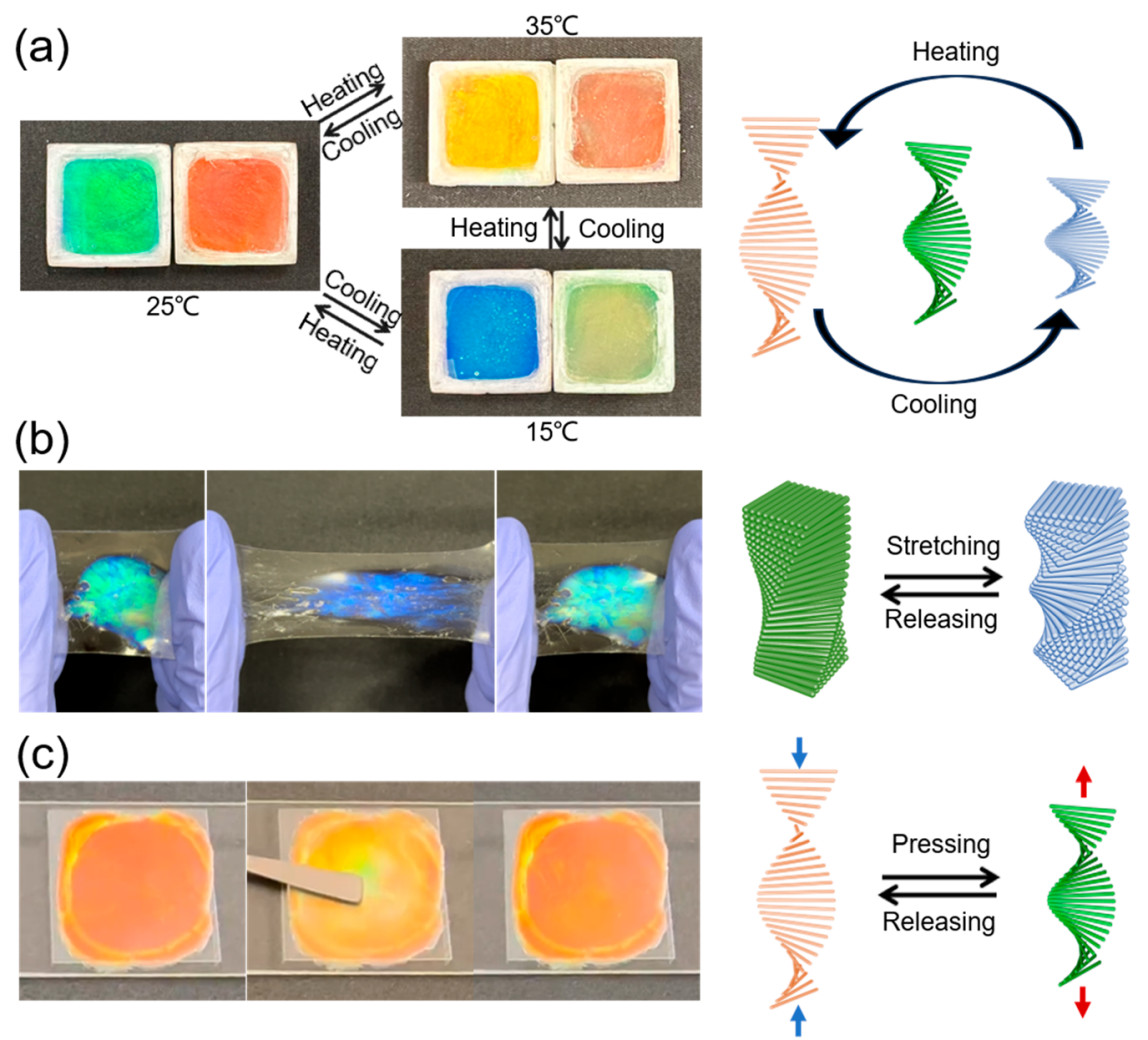
(a) Reversible color response of HPC/TEG-DE and HPC/DEG-EEA gels (left and right side of each photograph) subjected to continuous cycling between 15, 25, and 35 °C. (b) Reversible structural color change of HPC/TEG-DE under stretching and releasing. (c) Reversible structural color change of HPC/DEG-EEA under pressing and releasing. The right side of each figure corresponds to the schematic diagrams of reversible structural changes of HPC periodicity under different environmental stimuli.

By manipulating the degree of PEG unsaturation, the HPC/PEG gel exhibits the capability to span the complete RGB (red, green, and blue) spectrum. This excellent color controllability, coupled with the gel’s inherent flexibility, facilitates our exploration of its utility as a sensor, encompassing applications such as temperature, tension, and pressure sensors. The primary objective of our study is to investigate the gel’s responsiveness, sensitivity, and reversibility when subjected to various stimuli. Toward this goal, we first conducted temperature tests on two distinct gel formulations. From 25 to 35 °C, the HPC/TEG-DE gel exhibited a vivid green to yellow shift (Figure 6a). At 15 °C, the gel turned blue, and returning to 25 °C reinstated the green color. Excitingly, this reversible tuning occurred regardless of the temperature change order and start point. Testing HPC/DEG-EEA gels revealed the same red-shift with heating and a blue-shift with cooling. Thus, temperature reversibly expands and contracts the composite gel spacing, as exhibited in Figure 6a and Supporting Video S1. However, some minor, tolerable wavelength shifts can be observed from the CD spectrum. Consequently, the discrepancy observed between the calculated and measured resonance wavelengths may stem from inherent, permissible factors, such as nonuniformity of the gels and the exact temperature of the room when conducting the experiment. Furthermore, we noticed the same wavelength shifts in both POM measurements and CD experiments, which are in good agreement. This exemplary color reactivity and precise reversibility to temperature changes highlight the gels’ potential for tunable colorimetric temperature sensors and indicators. The mechanism enables a noncontact, self-reporting system for tracking real-time temperature fluctuations in residential, industrial, and biomedical applications. Moreover, the distinctive color responsiveness to slight temperature variations provides opportunities for high-resolution thermal mapping and detection devices. Overall, the simplistic sustainable wood-derived platform exhibits a promising functionality.

To evaluate the structural color response under deformation, a wearable strain sensor was developed using the HPC/TEG-DE gel encased in transparent rubber (Figure 6b and Supporting Video S2). Stretching causes the color to shift from green to blue due to the structure contraction and reflected wavelength change. Releasing the strain reverts the periodicity and color to the original state, showing fully reversible tuning. The application of pressure to HPC/DEG-EEA induces a discernible transition in its color, shifting from orange to yellow-green, and subsequently returning to its original orange hue upon release (see Figure 6c and Supporting Video S3). These reversible color alterations can be attributed to compression or expansion of the liquid crystal structure induced by pressure, as elucidated in the schematic on the right side of Figure 6. In summary, both temperature variation and mechanical manipulation of the cholesteric spacing give rise to reversible shifts in coloration. Upon consolidating these various phenomena, an observation of paramount significance is the enduring color stability exhibited by the gels even subsequent to undergoing numerous cycles of reversible stimulations.

It is crucial to underscore the consistent homogeneity in the coloration of all composite gels, both prior to and following exposure to environmental stimuli. This unequivocally demonstrates the effectiveness of PEG doping in the context of HPC gel applications. This assertion finds support in one of the microscopic sections within the SEM images (Figure 3e–h), where the microstructure of HPC composite membranes exhibits a notably enhanced regularity following the addition of PEGs. This heightened regularity can be directly attributed to the polarizing effect of PEGs on the alignment of the HPC cholesteric phase. Another noteworthy advantage is that the incorporation of PEGs broadens the range of color variation for HPC gels in response to environmental conditions. While the color shift range for pure HPC gels spans from blue-green to colorless, for HPC/TEG-DE, HPC/DEG-EEA, and HPC/DEG-DA, it extends from yellow-green, orange, and red to colorless, respectively. This enhanced regularity can be unequivocally attributed to the influence exerted by PEG’s polarization on the ordering of the HPC cholesteric phase. Overall, the manipulation of PEG polarization stands as a critical factor in facilitating multifunctional sensing capabilities within the composite gel. This exemplary multifunctional capability highlights the significant potential of these sustainable cellulose-based materials for smart devices. The intricate colorimetric responsiveness to strain promises application in biocompatible skin-attachable sensors for real-time health monitoring and human–machine interfaces. Moreover, the distinctive pressure-induced color variations provide opportunities for interactive touch/stress sensing technologies. Overall, the simplistic natural-wood-derived platform exhibits impressive dynamic optical properties that are suited for next-generation sensors and displays.

## Conclusions

In summary, our research has successfully yielded innovative fixed-color organic photonic gels, demonstrating their potential as effective colorimetric sensors. A key discovery in our work is the identification of an interesting mechanism for adjusting the internal nanostructure of these gels. This is achieved through the incorporation of PEGs with specially designed end-group polarities. This work shows that the reflectance spectrum of hydroxypropyl cellulose gels can be controlled by adjusting the degree of unsaturation in PEGs. Importantly, this process does not alter the chiroptical properties, namely, the inherent handedness of the gel’s structure. Furthermore, these gels exhibit dynamic and reversible color changes in response to various stimuli, such as temperature fluctuations and applied tensile forces, highlighting their versatility.

Notably, the introduction of PEGs with varying polarities not only enhances color uniformity but also expands the spectrum of color variations achievable within the HPC composite gels. This feature adds functionality to our photonic gels and makes them highly adaptable for diverse applications. The gels’ ability to self-assemble into liquid crystal structures in highly viscoelastic systems, without the need for precise helical alignment, offers a special platform for investigating solvent-dependent kinetic processes. Moreover, the inherent moldability of these gels provides possibilities for creating structurally colored materials with customizable shapes, making them well-suited for potential applications in wearable technology.

A significant advantage of these cellulose-derived gels over conventional synthetic thermotropic systems lies in their sustainable and nontoxic nature, addressing both economic and environmental concerns. Our findings not only contribute to a deeper understanding of the mechanisms behind dynamic structural color tuning but also showcase the practicality of a simple, sustainable, and cellulosic material. This work presents a pathway for the development of smart multifunctional technologies that leverage the stretchability and reversible optical properties of these organic photonic gels.

## Methods and Experimental Details

### Chemicals and Materials

Hydroxypropyl cellulose (HPC) was supplied by NIPPON SIDA CO., LTD, with a relative molecular weight (*M*_w_) of 40000. Tetraethylene glycol dimethyl ether (TEG-DE, *M*_w_ = 222), diethylene glycol ethyl ether acrylate (DEG-EEA, *M*_w_ = 188), and diethylene glycol diacrylate (DEG-DA, *M*_w_ = 214) were purchased from Sigma-Aldrich Co. Ltd. Hydrochloric acid (HCl, 37%) and glutaraldehyde were purchased from Fisher Scientific. All remaining chemicals were of analytical grade and were employed without any additional purification steps.

### Preparation of HPC/PEG Gels

Water (2.35 g) was vigorously mixed with HPC (5.00 g) using a Mixer centrifugal stirrer (3500 rpm, 5 min) to produce an initial HPC solution of 68 wt %. The different end-group PEGs (TEG-DE, DEG-EEA, and DEG-DA) were each added to the HPC solution at 5 wt % (relative to the mass of HPC) and dispersed as before (3500 rpm, 5 min).

### Optical Characterization of HPC/PEG Gels

1 g of HPC or HPC/PEG gel was spread and encapsulated between two glass slides. The reflectance values of the gels were recorded using a UV–vis spectrophotometer (Shimadzu UV-1900, Japan) in the wavelength range of 400–700 nm. Three spectroscopic measurements were taken at different locations across each sample and averaged.

### Polarized Optical Microscopy (POM)

White light was generated by a tungsten lamp (Luxte fiber optic light source universal series 1300). The light was collimated by 2 lenses (Thorlab) and 2 irises (Thorlab), which formed a 4f system. Then it passed the first linear polarizer. A 10× objective lens (Olympus) focused the light on the sample and then collected the reflected light. A beam splitter directed the reflected light to the collection path of the setup. An orthogonal linear polarizer was placed before a CCD camera (Lumenera) that captured the POM image.

### Circular Dichroism (CD)

A laser beam with a tunable wavelength from 400 to 800 nm was produced by a Coherent Chameleon Compact OPO laser system. The laser was collimated by 2 lenses and 2 irises, which formed a 4f system. It passed through a quarter waveplate (Thorlab) to generate left- and right-handed circularly polarized light. Then it was focused on the sample with a convex lens (focal length of 50 mm, Thorlab) and collected with the same lens. A beam splitter directed the reflected light to the collection path of the setup, and a spectrometer (Horiba iHR550) was used to obtain the spectra. All experiments were conducted twice separately for left- and right-handed circularly polarized light. The CD spectra were obtained by using eq 2.

### Zeta Potential

The HPC and HPC/PEG solutions were prepared separately, keeping the HPC concentration at 0.05 wt %, where the mass ratio of HPC to PEG was constant. The zeta potentials of the HPC and PEG composite solutions were measured with a Zetasizer Nano instrument (ZS-90, Malvern Instruments, U.K.). All measurements were conducted by using freshly prepared samples and were then averaged across three instances.

### Scanning Electron Microscopy (SEM)

In order to observe the internal structure of HPC/PEG, the gel was cured into a thin film by using glutaraldehyde cross-linking. Glutaraldehyde (0.3 g) was first diluted in aqueous HCl (0.5 M, 2.1 g). This acidic glutaraldehyde solution was subsequently added to dry HPC (5.0 g) and immediately mixed with a planetary centrifuge at 3500 rpm for 5 min. Mixing was then continued in a planetary centrifuge with or without the addition of PEGs (TEG-DE, DEG-EEA, or DEG-DA) under the same conditions (3500 rpm for 5 min). This was followed by degassing via centrifugation at 4000*g* for 1 h. Finally, the gel was coated on a glass slide with a Doctor Blade Coater (1.5 mm spacing) and placed in an oven at 70 °C for 2 h to obtain HPC/PEG films. The surface and cross-sectional morphologies of the sputter-coated crystalline films were observed using a field emission scanning electron microscope (SEM, S3700 Hitachi Ltd. Japan) at an accelerating voltage of 10 kV. The determination of layer spacing within the composite film was conducted with Nano Measure software, with a minimum of 50 measurements taken to calculate the average value.

### Applications

#### Temperature Response Testing

HPC/TEG-DE and HPC/DEG-EEA gels were selected to evaluate the color responses to temperature changes. The gels were sandwiched between the slides and sealed with Parafilm. They were imaged with a camera at 15, 25, and 35 °C to assess color shifts.

#### Mechanical Stretching Evaluation

An HPC/TEG-DE gel was encapsulated in transparent double-sided tape and subjected to stretching and release while the color changed.

#### Mechanical Compression Evaluation

An HPC/DEG-EEA gel coated on slides and sealed with Parafilm underwent stepwise compression and pressure release while imaging color alterations.

## Supporting Information Available

Color change of composite gel under changed temperature (MP4)
Color change of composite gel under stretching and releasing (MP4)
Color change of composite gel under pressing and releasing (MP4)
Additional data on materials characterization, experimental methods, and analysis (PDF)

## References

[ref1] Ito M. M.; Gibbons A. H.; Qin D.; Yamamoto D.; Jiang H.; Yamaguchi D.; Tanaka K.; Sivaniah E. Structural Colour Using Organized Microfibrillation in Glassy Polymer Films. Nature 2019, 570 (7761), 363–367. 10.1038/s41586-019-1299-8. 31217598

[ref2] Goodling A. E.; Nagelberg S.; Kaehr B.; Meredith C. H.; Cheon S. I.; Saunders A. P.; Kolle M.; Zarzar L. D. Colouration by Total Internal Reflection and Interference at Microscale Concave Interfaces. Nature 2019, 566 (7745), 523–527. 10.1038/s41586-019-0946-4. 30814712

[ref3] Tadepalli S.; Slocik J. M.; Gupta M. K.; Naik R. R.; Singamaneni S. Bio-Optics and Bio-Inspired Optical Materials. Chem. Rev. 2017, 117 (20), 12705–12763. 10.1021/acs.chemrev.7b00153. 28937748

[ref4] Kim J. B.; Chae C.; Han S. H.; Lee S. Y.; Kim S.-H. Direct Writing of Customized Structural-Color Graphics with Colloidal Photonic Inks. Sci. Adv. 2021, 7 (48), eabj8780 10.1126/sciadv.abj8780. 34818030 PMC8612532

[ref5] Xu M.; Wu X.; Yang Y.; Ma C.; Li W.; Yu H.; Chen Z.; Li J.; Zhang K.; Liu S. Designing Hybrid Chiral Photonic Films with Circularly Polarized Room-Temperature Phosphorescence. ACS Nano 2020, 14 (9), 11130–11139. 10.1021/acsnano.0c02060. 32813496

[ref6] Shu F.-Z.; Yu F.-F.; Peng R.-W.; Zhu Y.-Y.; Xiong B.; Fan R.-H.; Wang Z.-H.; Liu Y.; Wang M. Dynamic Plasmonic Color Generation Based on Phase Transition of Vanadium Dioxide. Adv. Opt. Mater. 2018, 6 (7), 1700939 10.1002/adom.201700939.

[ref7] Xiao M.; Hu Z.; Wang Z.; Li Y.; Tormo A. D.; Le Thomas N.; Wang B.; Gianneschi N. C.; Shawkey M. D.; Dhinojwala A. Bioinspired Bright Noniridescent Photonic Melanin Supraballs. Sci. Adv. 2017, 3 (9), e1701151 10.1126/sciadv.1701151. 28929137 PMC5600532

[ref8] Liu F.; Dong B. Q.; Liu X. H.; Zheng Y. M.; Zi J. Structural Color Change in Longhorn Beetles Tmesisternus Isabellae. Opt. Express 2009, 17 (18), 16183–16191. 10.1364/OE.17.016183. 19724618

[ref9] Whitney H. M.; Kolle M.; Andrew P.; Chittka L.; Steiner U.; Glover B. J. Floral Iridescence, Produced by Diffractive Optics, Acts As a Cue for Animal Pollinators. Science 2009, 323 (5910), 130–133. 10.1126/science.1166256. 19119235

[ref10] Wang S.; Lu A.; Zhang L. Recent Advances in Regenerated Cellulose Materials. Prog. Polym. Sci. 2016, 53, 169–206. 10.1016/j.progpolymsci.2015.07.003.

[ref11] Cheng Z.; Ma Y.; Yang L.; Cheng F.; Huang Z.; Natan A.; Li H.; Chen Y.; Cao D.; Huang Z.; Wang Y.; Liu Y.; Yang R.; Zhu H. Plasmonic-Enhanced Cholesteric Films: Coassembling Anisotropic Gold Nanorods with Cellulose Nanocrystals. Adv. Opt. Mater. 2019, 7 (9), 1801816 10.1002/adom.201801816.

[ref12] Cheng Z.; Ye H.; Cheng F.; Li H.; Ma Y.; Zhang Q.; Natan A.; Mukhopadhyay A.; Jiao Y.; Li Y.; Liu Y.; Zhu H. Tuning Chiral Nematic Pitch of Bioresourced Photonic Films via Coupling Organic Acid Hydrolysis. Adv. Mater. Interfaces 2019, 6 (7), 1802010 10.1002/admi.201802010.

[ref13] Godinho M. H.; Gray D. G.; Pieranski P. Revisiting (Hydroxypropyl) Cellulose (HPC)/Water Liquid Crystalline System. Liq. Cryst. 2017, 44 (12-13), 2108–2120. 10.1080/02678292.2017.1325018.

[ref14] XUE C.; YU G.; HIRATA T.; TERAO J.; LIN H. Antioxidative Activities of Several Marine Polysaccharides Evaluated in a Phosphatidylcholine-Liposomal Suspension and Organic Solvents. Biosci. Biotechnol. Biochem. 1998, 62 (2), 206–209. 10.1271/bbb.62.206. 9532776

[ref15] Werbowyj R. S.; Gray D. G. Liquid Crystalline Structure In Aqueous Hydroxypropyl Cellulose Solutions. Mol. Cryst. Liq. Cryst. 1976, 34 (4), 97–103. 10.1080/15421407608083894.

[ref16] Skinner G. W.; Harcum W. W.; Barnum P. E.; Guo J.-H. The Evaluation of Fine-Particle Hydroxypropylcellulose as a Roller Compaction Binder in Pharmaceutical Applications. Drug Dev. Ind. Pharm. 1999, 25 (10), 1121–1128. 10.1081/DDC-100102278. 10529893

[ref17] Kawashima Y.; Takeuchi H.; Hino T.; Niwa T.; Lin T.-L.; Sekigawa F.; Kawahara K. Low-Substituted Hydroxypropylcellulose as a Sustained-Drug Release Matrix Base or Disintegrant Depending on Its Particle Size and Loading in Formulation. Pharm. Res. 1993, 10 (3), 351–355. 10.1023/A:1018975919598. 8464806

[ref18] Karki S.; Kim H.; Na S.-J.; Shin D.; Jo K.; Lee J. Thin Films as an Emerging Platform for Drug Delivery. Asian J. Pharm. Sci. 2016, 11 (5), 559–574. 10.1016/j.ajps.2016.05.004.

[ref19] Mariotti M.; Pagani M. A.; Lucisano M. The Role of Buckwheat and HPMC on the Breadmaking Properties of Some Commercial Gluten-Free Bread Mixtures. Food Hydrocoll. 2013, 30 (1), 393–400. 10.1016/j.foodhyd.2012.07.005.

[ref20] Onogi Y.; White J. L.; Fellers J. F. Structural Investigations of Polymer Liquid-Crystalline Solutions: Aromatic Polyamides, Hydroxy Propyl Cellulose, and Poly(γ-Benzyl-L-Glutamate). J. Polym. Sci. Polym. Phys. Ed. 1980, 18 (4), 663–682. 10.1002/pol.1980.180180401.

[ref21] Zhang Z.; Chen Z.; Wang Y.; Zhao Y. Bioinspired Conductive Cellulose Liquid-Crystal Hydrogels as Multifunctional Electrical Skins. Proc. Natl. Acad. Sci. U. S. A. 2020, 117 (31), 18310–18316. 10.1073/pnas.2007032117. 32675247 PMC7414159

[ref22] Li D.; Wu J.-M.; Liang Z.-H.; Li L.-Y.; Dong X.; Chen S.-K.; Fu T.; Wang X.-L.; Wang Y.-Z.; Song F. Sophisticated yet Convenient Information Encryption/Decryption Based on Synergistically Time-/Temperature-Resolved Photonic Inks. Adv. Sci. 2023, 10 (5), 2206290 10.1002/advs.202206290. PMC9929127 36504335

[ref23] Charlet G.; Gray D. G. Solid Cholesteric Films Cast from Aqueous (Hydroxypropyl)Cellulose. Macromolecules 1987, 20 (1), 33–38. 10.1021/ma00167a007.

[ref24] Stumpel J. E.; Gil E. R.; Spoelstra A. B.; Bastiaansen C. W. M.; Broer D. J.; Schenning A. P. H. J. Stimuli-Responsive Materials Based on Interpenetrating Polymer Liquid Crystal Hydrogels. Adv. Funct. Mater. 2015, 25 (22), 3314–3320. 10.1002/adfm.201500745.

[ref25] Wei J.; Aeby X.; Nyström G. Printed Structurally Colored Cellulose Sensors and Displays. Adv. Mater. Technol. 2023, 8 (1), 2200897 10.1002/admt.202200897.

[ref26] Andersson H.; Hjärtstam J.; Stading M.; von Corswant C.; Larsson A. Effects of Molecular Weight on Permeability and Microstructure of Mixed Ethyl-Hydroxypropyl-Cellulose Films. Eur. J. Pharm. Sci. 2013, 48 (1), 240–248. 10.1016/j.ejps.2012.11.003. 23159668

[ref27] Barty-King C. H.; Chan C. L. C.; Parker R. M.; Bay M. M.; Vadrucci R.; De Volder M.; Vignolini S. Mechanochromic, Structurally Colored, and Edible Hydrogels Prepared from Hydroxypropyl Cellulose and Gelatin. Adv. Mater. 2021, 33 (37), 2102112 10.1002/adma.202102112. PMC11468689 34323315

[ref28] Vshivkov S. A.; Adamova L. V.; Rusinova E. V.; Safronov A. P.; Dreval’ V. E.; Galyas A. G. Thermodynamics of Liquid-Crystalline Solutions of Hydroxypropyl Cellulose in Water and Ethanol. Polym. Sci. Ser. A 2007, 49 (5), 578–583. 10.1134/S0965545X07050124.

[ref29] Nishio Y.; Chiba R.; Miyashita Y.; Oshima K.; Miyajima T.; Kimura N.; Suzuki H. Salt Addition Effects on Mesophase Structure and Optical Properties of Aqueous Hydroxypropyl Cellulose Solutions. Polym. J. 2002, 34 (3), 149–157. 10.1295/polymj.34.149.

[ref30] Ebers L.-S.; Laborie M.-P. Direct Ink Writing of Fully Bio-Based Liquid Crystalline Lignin/Hydroxypropyl Cellulose Aqueous Inks: Optimization of Formulations and Printing Parameters. ACS Appl. Bio Mater. 2020, 3 (10), 6897–6907. 10.1021/acsabm.0c00800. 35019351

[ref31] Infanger S.; Haemmerli A.; Iliev S.; Baier A.; Stoyanov E.; Quodbach J. Powder Bed 3D-Printing of Highly Loaded Drug Delivery Devices with Hydroxypropyl Cellulose as Solid Binder. Int. J. Pharm. 2019, 555, 198–206. 10.1016/j.ijpharm.2018.11.048. 30458260

[ref32] Walters C. M.; Boott C. E.; Nguyen T.-D.; Hamad W. Y.; MacLachlan M. J. Iridescent Cellulose Nanocrystal Films Modified with Hydroxypropyl Cellulose. Biomacromolecules 2020, 21 (3), 1295–1302. 10.1021/acs.biomac.0c00056. 32053370

[ref33] Younas M.; Noreen A.; Sharif A.; Majeed A.; Hassan A.; Tabasum S.; Mohammadi A.; Zia K. M. A Review on Versatile Applications of Blends and Composites of CNC with Natural and Synthetic Polymers with Mathematical Modeling. Int. J. Biol. Macromol. 2019, 124, 591–626. 10.1016/j.ijbiomac.2018.11.064. 30447361

[ref34] Lee S.-Y.; Chun S.-J.; Kang I.-A.; Park J.-Y. Preparation of Cellulose Nanofibrils by High-Pressure Homogenizer and Cellulose-Based Composite Films. J. Ind. Eng. Chem. 2009, 15 (1), 50–55. 10.1016/j.jiec.2008.07.008.

[ref35] Rey A. D. Liquid Crystal Models of Biological Materials and Processes. Soft Matter 2010, 6 (15), 3402–3429. 10.1039/B921576J.

[ref36] Ho R.-M.; Chiang Y.-W.; Lin S.-C.; Chen C.-K. Helical Architectures from Self-Assembly of Chiral Polymers and Block Copolymers. Prog. Polym. Sci. 2011, 36 (3), 376–453. 10.1016/j.progpolymsci.2010.09.001.

[ref37] Yashima E.; Maeda K.; Iida H.; Furusho Y.; Nagai K. Helical Polymers: Synthesis, Structures, and Functions. Chem. Rev. 2009, 109 (11), 6102–6211. 10.1021/cr900162q. 19905011

[ref38] Dey J.; O’Donoghu A. C.; More O’Ferrall R. A. Equilibrium Constants for Dehydration of Water Adducts of Aromatic Carbon-Carbon Double Bonds. J. Am. Chem. Soc. 2002, 124 (29), 8561–8574. 10.1021/ja0126125. 12121097

[ref39] Carey F. A.; Sundberg R. J. Reduction of Carbon-Carbon Multiple Bonds, Carbonyl Groups, and Other Functional Groups. In Advanced Organic Chemistry: Part B: Reactions and Synthesis; Carey F. A., Sundberg R. J., Eds.; Springer US: 2007; pp 367–471. 10.1007/978-0-387-71481-3_5.

[ref40] Silverman M. P.; Badoz J.; Briat B. Chiral Reflection from a Naturally Optically Active Medium. Opt. Lett. 1992, 17 (12), 886–888. 10.1364/OL.17.000886. 19794663

