# Supplementary material for: Sustainable Cellulose-Derived Organic Photonic Gels with Tunable and Dynamic Structural Color
Source: ACS Nano. 2024 Jan 12;18(4):3627–35. doi: 10.1021/acsnano.3c11432 (PMC10832026; doi:10.1021/acsnano.3c11432)
Supplement: Supplementary file 4 — nn3c11432_si_004.pdf [file nn3c11432_si_004.pdf]

Supporting Information

Sustainable Cellulose-Derived Organic Photonic Gels with Tunable and Dynamic Structural Color

Luyao Huang[1], Xianzhe Zhang[2], Lin Deng[2], Ying Wang[1], Yongmin Liu[1, 2, *], Hongli Zhu[1, *]

[1]Department of Mechanical and Industrial Engineering, Northeastern University, 360 Huntington Avenue, Boston, Massachusetts 02115, United States

[2]Department of Electrical and Computer Engineering, Northeastern University, 360 Huntington Avenue, Boston, Massachusetts 02115, United States

* Corresponding author: Hongli Zhu. E-mail: h.zhu@northeastern.edu; Yongmin Liu: y.liu@northeastern.edu

Results and Discussion

Table S1. The uniformity characterization of different HPC gels

	HPC	HPC/TEG-DE	HPC/DEG-EEA	HPC/DEG-DA
Wavelength difference (nm)	7.0	2.5	6.0	4.5
Standard deviation	2.9	1.0	2.5	1.8

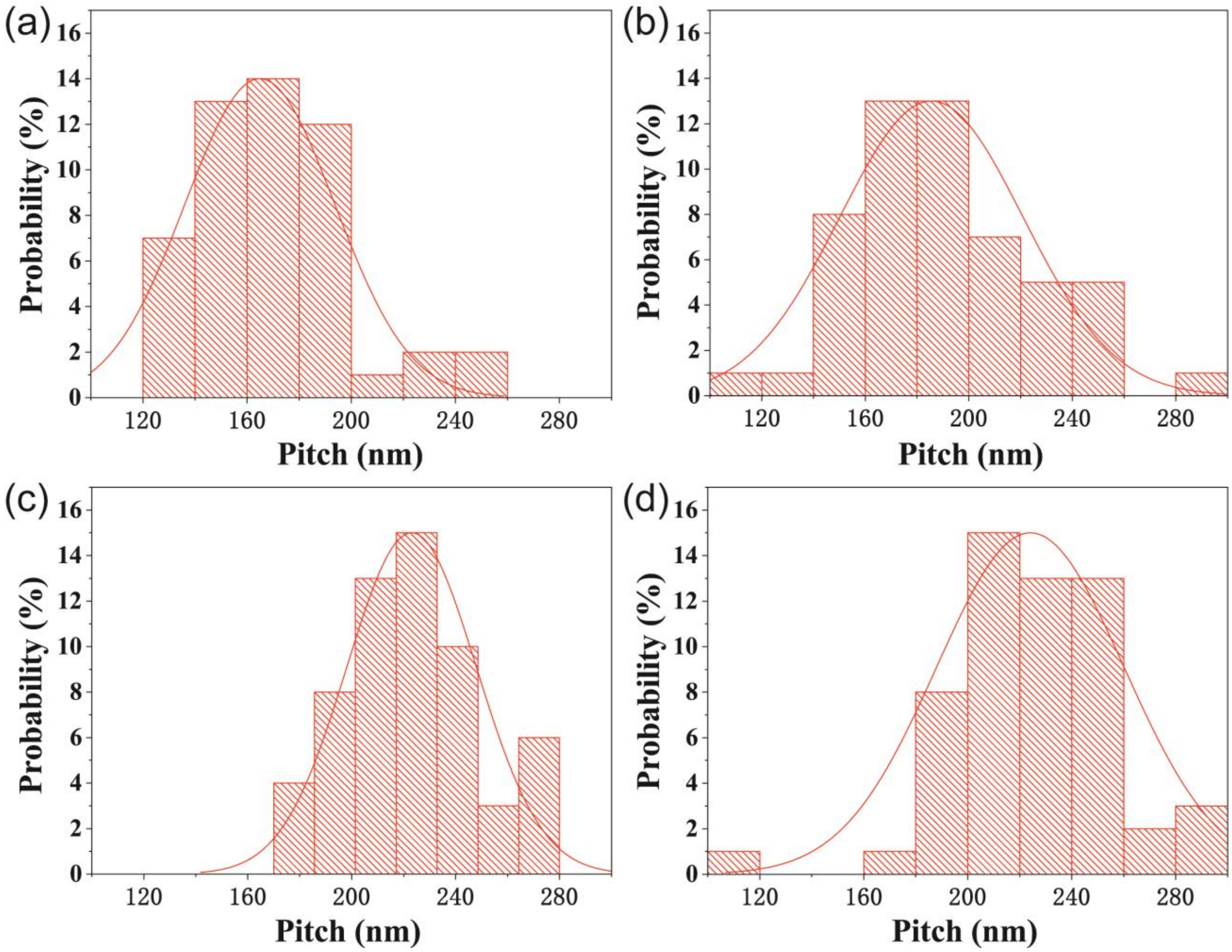

Figure S1. The distribution of pitches for four different films, namely (a) HPC, (b) HPC/TEG-DE, (c) HPC/DEG-EEA, and (d) HPC/DEG-DA, was statistically analyzed from the SEM images.

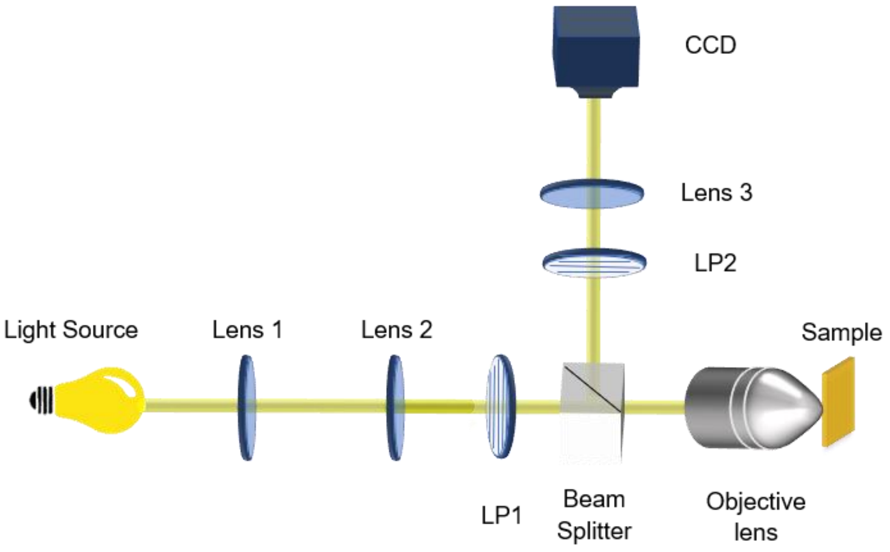

Figure S2. Schematic diagram of the optical setup for polarized optical microscopy.

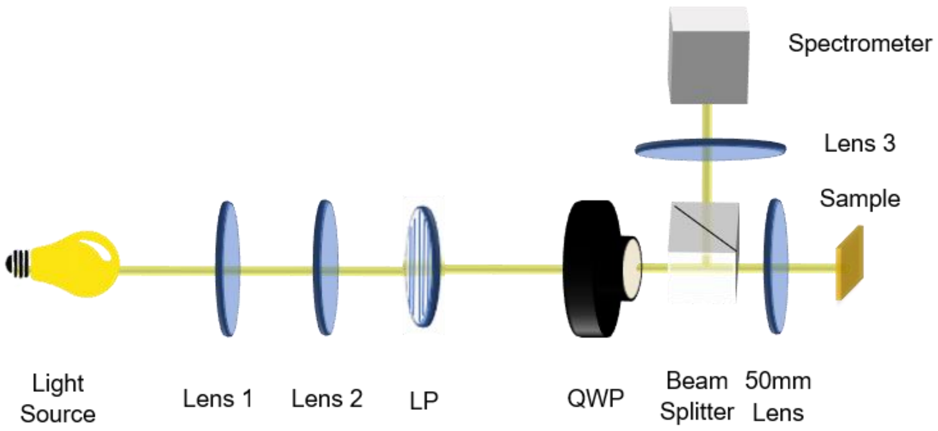

Figure S3. Schematic diagram of the optical setup for circular dichroism measurement.

Video S1. Color change of composite gel under changed temperature (shown in the attachment)

Video S2. Color change of composite gel under pressing and releasing (shown in the attachment)

Video S3. Color change of composite gel under stretching and releasing (shown in the attachment)
